# Small Heterocyclic Ligands as Anticancer Agents: QSAR with a Model G-Quadruplex

**DOI:** 10.3390/molecules27217577

**Published:** 2022-11-04

**Authors:** Jose Kaneti, Vanya Kurteva, Milena Georgieva, Natalia Krasteva, George Miloshev, Nadezhda Tabakova, Zhanina Petkova, Snezhana M. Bakalova

**Affiliations:** 1 Institute of Organic Chemistry with Centre of Phytochemistry, Bulgarian Academy of Sciences, 1113 Sofia, Bulgaria; 2 Institute of Molecular Biology “R. Tsanev”, Bulgarian Academy of Sciences, 1113 Sofia, Bulgaria; 3 Institute of Biophysics and Biomedical Engineering, Bulgarian Academy of Sciences, 1113 Sofia, Bulgaria

**Keywords:** nitrogen heterocyclic ligands, G-quadruplexes, stacking interactions, DFT GQ-ligand affinity vs. IC50 relationship, quantum chemical calculations

## Abstract

G-quadruplexes (GQs) have become valid targets for anticancer studies in recent decades due to their multifaceted biological function. Herewith, we aim to quantify interactions of potential heterocyclic ligands (Ls) with model GQs. For seven 4-aminoquinazolines and three 2-heteroaryl perimidines, seven of this ten-membered group so far unknown, we use routine quantum chemical modeling. As shown in the literature, a preferred mode of interaction of heterocycles with cellular structures is stacking to exposable faces of G-quadruplexes. To exploit the energy of this interaction as a molecular descriptor and achieve the necessary chemical precision, we use state of the art large-scale density functional theory (DFT) calculations of stacked heterocycles to a GQ. Actually, the GQ has been simplified for the computation by stripping it off all pentose phosphate residues into a naked model of stacked guanine quartets. The described model thus becomes computable. The obtained heterocyclic ligand GQ.L stacking energies, that is, their GQ affinities, are the necessary ligand descriptors. Using the ligand biological inhibitory activities (IC_50_) on a human malignant melanoma A375 cell line, we obtain a good linear relationship between computed ligand stacking affinities to GQ, and experimental log (IC_50_) values. Based on the latter relationship, we discuss a putative mechanism of anticancer activity of heterocyclic ligands via stacking interactions with GQs and thereby controlling cell regulatory activity. This mechanism may tentatively be applied to other condensed five- and six-membered small heterocycles as well.

## 1. Introduction

Alkaloids and their chemical analogs have long been among the most popular and sought organic natural, laboratory, and industrial products for a leading reason—their beneficial physiological activity on human health [1]. Recently, their activity and applications have increasingly been related to their capability to interact with a particular category of nucleic acids (NAs)—the four-stranded G-quadruplexes [1]. While not directly involved in preserving and transferring genetic information, G-quadruplexes have been disclosed as decisive participants in a plethora of cellular processes such as NA biosynthesis, replication, transcription, oncogenesis, etc. Telomeres are known sites accumulating G-quadruplexes, which are essential to their functioning in cell reproduction, aging, genetic stability, and cancer. A G-quadruplex may inhibit telomerase activity, directly affecting cancer cells and primary tumors [2]. A G-quadruplex may dissociate telomere-binding proteins, thus leading to dysfunction and, finally, to apoptosis or senescence [3]. A G-quadruplex interferes with telomeric replication by impairing replication fork progression [4]. Thus, knowledge of ligand structures stabilizing G-quadruplexes would allow for the specific design of heterocyclic systems targeting cancer cell function [1,5].

The recent decade has seen a number of efforts to quantify the anticancer activities of a series of selected heterocycles on cultivated cancer cell cultures [1,6,7]. The results of these efforts outline significant structure-activity trends in a series of quinazoline derivatives [6], indeno-isoquinoline derivatives, specifically on an isolated MYC-cancer promoter [7], and more generally in G-quadruplexes of various functions, structures, and sizes, as well as quadruplex targeting heterocyclic ligands [8,9,10]. Conversely, the belief that G4-ligands lack selectivity due to targeting multiple quadruplexes and, thus, many different sites in the genome still has an important place in the literature [9]. This requires additional efforts to reduce the effects of variable binding of G4-ligands [9] and references therein), which remain attractive therapeutic agents nevertheless [10,11,12,13]. Moreover, one might consider a G-quadruplex itself as determining selectivity and attracting whatever (larger size) heterocycles to stack to its large G4 plane. In these terms, G-quadruplex selectivity toward crescent-shaped planar ligand chromophores has repeatedly been noticed [1,8] and exploited in search of novel anticancer heterocycles [13], even though the terms G-quadruplex and mechanism of action have not been mentioned together in the latter review [13]. The pressing demand for all studies of potential anticancer activity is the generalization of their biochemical pharmacology data in the form of IC_50_, their structure-dependent activity information, into the quantitative form [14]. For this purpose, understanding the IC_50_ of a ligand as its inhibition constant [15] should possess a value exponentially dependent on its G4 affinity. The latter quantity is computable theoretically.

We have ventured into the field while discussing the mechanism of biological activity of some quinazoline derivatives [16]. The latter has inevitably introduced us to the possible involvement of G-quadruplexes in our problem and the necessity to bring up adequate computational methodologies to its solution. Traditional molecular mechanics MM and molecular dynamics MD approaches do not seem capable of bringing sufficiently accurate results for G-quadruplex structures [17]. The problem is related to insufficient intrinsic computing accuracy and numerical noise developing with slow energy convergence for polyatomic structures of the size of G-quadruplexes [17]. The necessary theoretical and computational accuracy only looks achievable using large-scale quantum chemical calculations [17,18]. To reduce the computational problem to reasonable limits and improve accuracy as much as possible, we strip our G-quadruplex model off all nucleotide residues [16]. This leaves the model a column of stacked guanine tetrads with a central channel containing the pertinent stabilizing K^+^ or Na^+^ ions [8]. With a size of 130 to 260 and more atoms, the core G4-system is relatively easily amenable to quantum chemical calculations using density functional theory, DFT [19,20]. Improved “chemical precision” molecular orbital, MO, computations are also feasible [21,22].

## 2. Results

### 2.1. Synthesis

The studied ligands involve seven novel and three known compounds representing two groups of heterocycles, 4-aminoquinazolines and 2-heteroaryl perimidines, summarized in Table 1. Aminoquinazolines are synthesized from the parent 4-quinazolinone via a two-step protocol. The intermediate chlorides are obtained according to a literature procedure [22] and are used in the second step without purification to avoid decomposition. The conditions are optimized, and pure compounds are isolated in moderate to excellent yields depending on the amine reactivity. Perimidines are prepared from 1,8-diaminonaphthalene and a heteroaromatic aldehyde via a two-step, one-pot protocol [23]. The conditions are optimized, and pure compounds are isolated in moderate to excellent yields depending on the amine reactivity. Perimidines are prepared from 1,8-diaminonaphthalene and a heteroaromatic aldehyde via a two-step, one-pot protocol [24].

### 2.2. Biological Evaluation of the Effect of the Studied Heterocyclic Ligands

**Figure 1:**
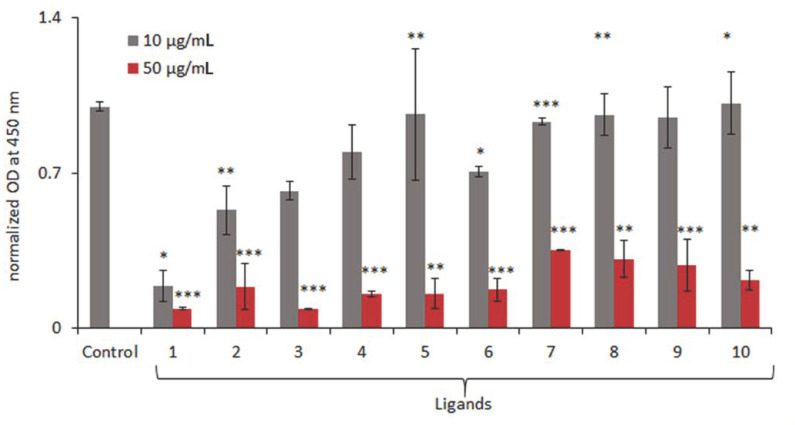
Cell viability analysis assessed by WST-1 assay. A375 melanoma cells are treated with small heterocyclic ligands with 10 and 50 μg/mL concentrations for 24 hours. Data are normalized to untreated cells and represent mean OD values ± s.d. from triplicate experiments. Data are statistically evaluated, and *, **, and *** denote *p* < 0.05, *p* < 0.01 and *p* < 0.001, respectively, compared with the control.

The cytotoxic activity of the studied heterocyclic ligands has been evaluated in vitro by assessing their cytotoxicity on melanoma cancer cells (A375 cell line). Cell viability has been estimated after 24 h of incubation with ligands. The cytotoxicity potential has been examined using several concentrations in the range 5–200 μg/mL. The obtained results for concentrations of 10 and 50 μg/mL. (Figure 1) have shown that the treatment of A375 cells with five of the ligands, 5, 7, 8, 9, and 10, with the lower concentration of 10 μg/mL slightly decreased cellular viability (between 3% and 7%). In comparison, the higher concentration of 50 μg/mL has significantly suppressed cell viability—up to 84%. At the low concentration, two other ligands, 4 and 6, have reduced cell viability by 21 and 29%, whereas at the high concentration, the reduction has been 71 and 83%, respectively. Ligands 2 and 3 have induced 46% and 38% inhibition of cell viability at the low concentration, and 81% and 92% at the high concentration. The most potent cytotoxicity effect on A375 melanoma cells has been demonstrated by ligand 1 with an 81% reduction at the low concentration and 91% at the higher concentration (Figure 1).

The data obtained on cytotoxicity of the small heterocyclic ligands have been used for calculations of half-maximal inhibitory concentrations (IC_50_) summarized in Table 1. The results in Table 1 show that 2-quinolinyl-quinazolines **1**–**3** are the most active ligands within the series studied. A comparison between quinazolines with identical 4-amino substituents, tetrahydroisoquinolinyl entry 1 vs. entry 5) and morpholinyl (entry 2 vs. entry 8), indicates that replacement of the 2-methyl substituent with 2-quinolinyl leads to a significant reduction in IC_50_, by approximately an order of magnitude. Therefore, it can be suggested that the aromatic substituent at the 2-position is essential for the activity of the particular ligands, possibly completing the preferred crescent ligand shape [8,10] and in line with the recent suggestion that quinoline residues would amplify G4 affinity to the corresponding ligand [14].

### 2.3. Computational Modeling

We have chosen the simplest G-quadruplex model, consisting of two guanine quartet sandwich layers and a single stabilizing potassium ion in between [8,10]. With these definitions, the ligand affinity has the simple form of

**Figure 2:**
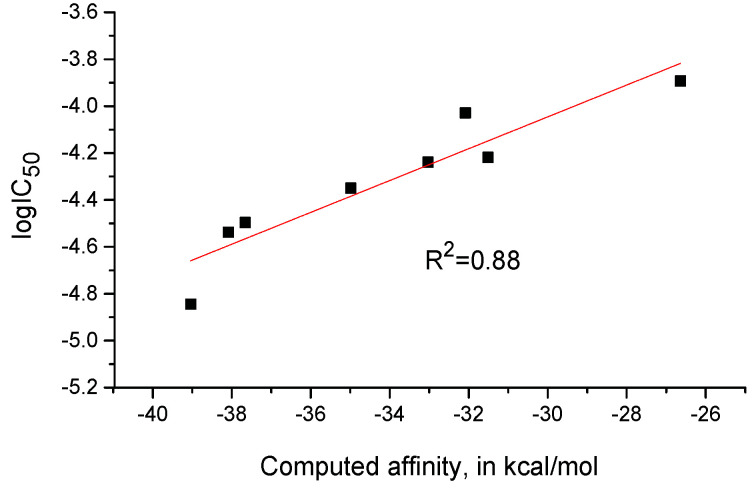
Plot of calculated wB97XD ligand affinities against log (IC_50_) for compounds **1–8**; R = 0.9357, R^2^ = 0.8756.

A_QL_ = E_QL_ − (E_Q_ + E_L_),

where E_QL_, E_Q_, and E_L_ are the computed total energies in vacuum for the quadruplex-ligand complex, free quadruplex, and free ligand, each completely optimized at the chosen theoretical level. An excerpt of the results is given in Table 1, and all data are summarized in Table S1. A plot of experimental IC_50_ values against computed ligand affinities is shown in Figure 2.

## 3. Discussion

**Scheme 1:**
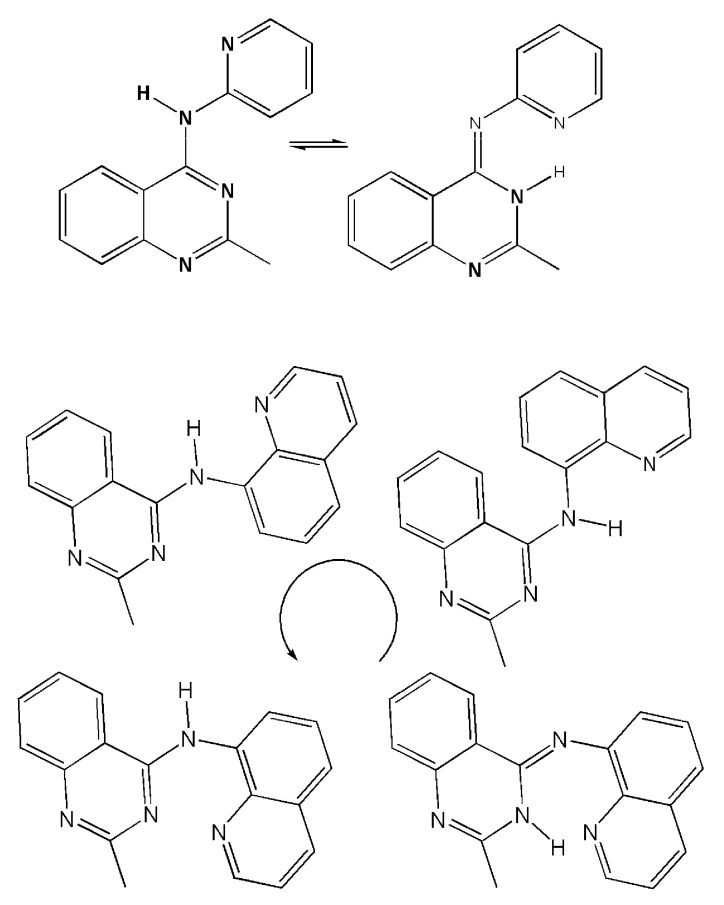
Tautomerism and rotational isomerization of N4-heterosubstituted quinazolines.

The clear trend revealed between experimental IC_50_ of studied small heterocyclic ligands indicates, at first sight, the good likeliness of the suggested stacking mechanism of their interactions with G-quadruplexes. Thus, the stacking of relatively small heterocyclic molecules is probably a valid interaction mechanism, apart from known modes of interaction with somewhat larger anticancer ligands targeting telomeres [16,24]. This suggestion does not eliminate different methods of attachment of small heterocycles to G4 quadruplexes, let alone branched and macrocyclic ligands [14]. The multidimensional problem of finding the minima of potential energy surfaces for these interactions has no unique solution, even from a purely mathematical viewpoint. Some optimism in this direction may be found in the earlier observation that molecular dynamics potential energy surfaces are relatively flat with deep global minima for bound ligands [25,26]. We may then focus on the structural properties of small ligands and the variations of their quadruplex interaction energies elicited by ligand characteristics. A case of deviations of interaction energy may arise from internal structural variations of a given ligand—the possibility for tautomeric forms and rotational isomerism. Examples of this point are given by tautomers of 4-amino quinazoline, with heterocyclic substituents at N_4_, 2-pyridyl, and 8-quinolyl, as shown in Scheme 1.

**Figure 3:**
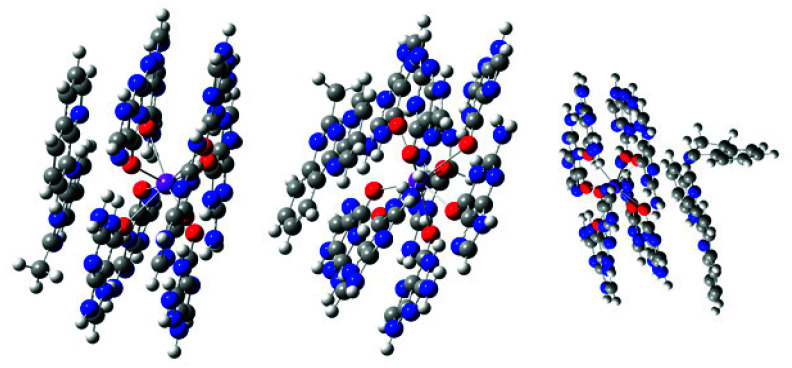
Complexes of model two-layered quadruplex Q2 with hetero-ligands, left: Q2 + **4**; center: Q2 + **9**, N3H tautomer; right: Q2 + **1**. In addition, see Figure S1 for opposite conformation of **1**.

The above intra-ligand processes may change the ligand affinities within a range of 0.5 to several kcal·mol^−1^. More serious changes are possible in cases where the ligand is non-planar and can attach with either its “concave” or “convex” side. At the same time, the overall energy changes of the G4-L complex energy remain within a couple of kcal·mol^−1^, these attachments may also induce changes in the shape of the G4-stack in the complex, see Figure 3. These ligand’s stacking and affinity variations may cause changes in the overall trend of proportionality of ligand affinities against quadruplex functioning, expressed in the above affinity against IC_50_ relationship. In the specific case of N4-2′-pyridyl and N4-8′-quinolyl substituents (entries 9 and 10 in Table 1), the compounds are, in fact, outliers to the generally linear relationship of affinities A_L_ to activities, log (IC_50_).

**Figure 4:**
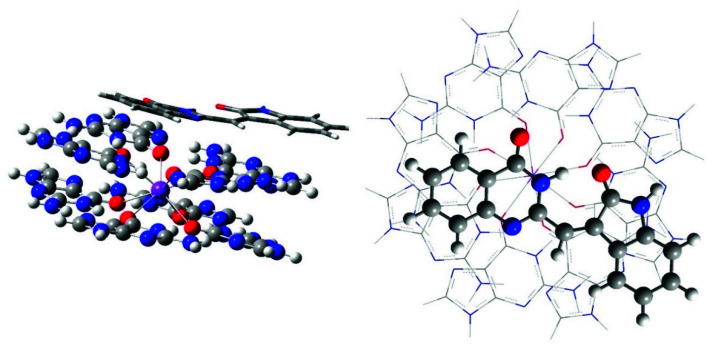
The adduct of schizocommunin, the rigid crescent shape on top, to the model quadruplex, optimized wB97XD/6-31G(d,p) geometry. Oxygen atoms are red, and nitrogen atoms are blue.

The demonstrated variability of ligand—G-quadruplex model interactions certainly takes place in their interactions, in reality, thus bringing some scattering into computed ligand affinities, also expressed in deteriorating the correlation coefficient of the relationship. The latter deterioration of the correlation is even more pronounced with calculated RI-MP2 ligand affinities, only with R = 0.56, where the geometries of ligand complexes are not optimized at the used level of theory. Thus, the apparent suggestion from Figure 3 is that higher ligand affinity to G4 is associated with planar, crescent-like structures **1** to **4** as frequently noted in earlier work [8,10]; see also Figure 4 and Figure S2. Here, we manage to quantify the intuitive trend into a structure-activity relationship at the wB97XD/6-31G(d,p) level of DFT theory [19,20] and remain convinced of the possibility of a more extensive selection of example heterocyclic molecules to yield better correlations of computed structural data, G4 stabilization affinities, against the experimental anticancer activity.

## 4. Materials and Methods

### 4.1. Synthesis

*General:* All reagents were purchased from Aldrich, Merck, and Fluka and used without further purification. The deuterated solvents were purchased from DeuteroGmbH. Fluka silica gel (TLC-cards 60778 with fluorescent indicator 254 nm) were used for TLC and R_f_-values determination. Merck Silica gel 60 (0.040–0.063 mm) (Darmstadt, Germany) was used for flash chromatography purification of the products. The melting points were determined in capillary tubes on SRS MPA100 OptiMelt (Sunnyvale, CA, USA) automated melting point system with a heating rate of 1 °C per min. The NMR spectra were recorded on Bruker Avance II+ 600 or NEO 400 spectrometers (Rheinstetten, Germany) in an appropriate solvent; the chemical shifts were quoted in ppm in δ-values against tetramethylsilane (TMS) as an internal standard, and the coupling constants were calculated in Hz. The assignment of the signals is confirmed by applying two-dimensional COSY, NOESY, HSQC, and HMBC techniques. The spectra were processed with the Topspin 3.6 program. The mass spectra were recorded in positive mode on Q Exactive Plus Hybrid Quadrupole-Orbitrap Mass Spectrometer Thermo Scientific (ESI HR-MS). The spectra are processed with Xcalibur Free Style version 4.5 (Thermo Fisher Scientific Inc., Waltham, MA, USA) program.

The studied ligands (Table 1) include seven novel (2–7 and 10) and three known (1, 8 and 9) compounds and can be divided into two series; aminoquinazolines and perimidines. Aminoquinazolines are synthesized from the corresponding quinazolinone via a two-step protocol. The intermediate chlorides are obtained according to a literature procedure [22,27,28] and are used in the second step without purification to avoid decomposition. The conditions are optimized, and pure compounds are isolated in moderate to excellent yields depending on the amine reactivity. Perimidines are prepared from 1,8-diaminonaphthalene and aromatic aldehyde via a two-step, one-pot protocol [23,29].

#### 4.1.1. Synthesis of 4-Chloroquinazolines

*4-chloro-2-methylquinazoline* was prepared according to a known procedure [22] from commercially available 2-methyl quinazoline-4(3H)-one. To a solution of 2-methyl quinazoline-4-one (3 mmol) and Et_3_N (5 mmol) in benzene (15 mL), POCl_3_ (4.5 mmol) was added, and the mixture was refluxed with stirring for 2.5 h. After cooling to room temperature, the reaction mixture was poured into ice water and was consecutively washed with aq. NaHCO_3_, brine, citric acid, brine, NaHCO_3_, and brine. The organic layer was dried over MgSO_4_ and evaporated to dryness to give the crude product, which was further used without purification.

*4-chloro-2-(2-quinolinyl)quinazoline* was prepared via a two-step protocol:

*Step 1.* A solution of anthranilamide (3 mmol), quinoline-2-carbaldehyde (3.3 mmol), and *p*-TsOH (0.15 mmol) in THF (25 mL) was stirred at room temperature for 2 h. Iodine (4.5 mmol) was then added, and the mixture was stirred at room temperature for 4 h. The products were partitioned between EtOAc and aq. Na_2_S_2_O_3_ solution. The organic layer was washed with brine and dried over MgSO_4_. The crude product was triturated with MeOH to give pure *2-(2-quinolynyl)quinazoline-4(3H)-one*: 59% yield; R_f_ 0.24 (DCM); m. p. 227.6–227.8 °C (lit. [16] 227.7–227.9 °C); ^1^H NMR (CDCl_3_) 7.555 (ddd, 1H, J 8.1, 7.1, 1.2, C*H*-6), 7.81–7.84 (m, 2H, C*H*-7 and C*H*-7 Q), 7.897 (ddd, 1H, J 8.1, 1.2, 0.5, C*H*-8), 7.922 (ddd, 1H, J 8.1, 1.3, 0.8, C*H*-5 Q), 8.182 (dd, 1H, J 8.4, 0.9, C*H*-8 Q), 8.376 (bd, 1H, J 8.5, C*H*-4 Q), 8.396 (ddd, 1H, J 7.9, 1.5, 0.5, C*H*-5), 8.678 (d, 1H, J 8.5, C*H*-3 Q), 11.229 (bs, 1H, N*H*); ^13^C NMR (CDCl_3_) 118.48 (*C*H-3 Q), 122.66 (*C*_q_-4a), 126.81 (*C*H-5), 127.60 (*C*H-6), 127.78 (*C*H-5 Q), 128.23 (*C*H-6 Q), 128.31 (*C*H-8), 129.32 (*C*_q_-4a Q), 129.69 (*C*H-8 Q), 130.54 (*C*H-7 Q), 134.64 (*C*H-7), 137.67 (*C*H-4 Q), 146.79 (*C*_q_-8a Q), 148.03 (*C*_q_-2 Q), 149.00 (*C*_q_-2), 149.09 (*C*_q_-8a), 161.44 (*C*=O).

*Step 2.* To a solution of 2-(2-quinolynyl)quinazoline-4-one (3 mmol) and Et_3_N (5 mmol) in benzene (15 mL) POCl_3_ (4.5 mmol) was added, and the mixture was refluxed with stirring for 2.5 h. After cooling to room temperature, the reaction mixture was poured into ice water and was consecutively washed with aq. NaHCO_3_, brine, citric acid, brine, NaHCO_3_, and brine. The organic layer was dried over MgSO_4_ and evaporated to dryness to give the crude product, which was further used without purification.

#### 4.1.2. Synthesis of 4-Aminoquinazolines

*General procedure:* A solution of crude 4-chloro-2-methylquinazoline or 4-chloro-2-(2-quinolynyl)quinazoline (2 mmol), amine (2.5 mmol) and Et_3_N (3 mmol) in ether, benzene or DCE (20 mL) was stirred at room temperature or at 100 °C in a closed vessel for 5–20 h. The reaction mixture was extracted with brine. The organic layer was dried over MgSO_4_ and purified by column choralography on silica gel.

For simplicity, the signals for quinoline nuclei are depicted as “Q” and those of substituent at the 4th position as “Pip”, “Mor”, “THQ”, “AP”, and “AQ” for piperidine, morpholine, tetrahydroquinoline, 2-aminopyridine, and 8-aminoquinoline, respectively.

*2-methyl-4-(morpholin-4-yl)quinazoline:* Conditions: ether, rt, 20 h; 94% yield (overall from two steps); R_f_ 0.27 (EtOAc); m. p. 267.6–267.9 °C as HCl salt (lit. [24] m. p. not given); ^1^H NMR (CDCl_3_) 2.695 (s, 3H, C*H_3_*), 3.782 (m, 4H, C*H_2_*-3 and C*H_2_*-5 Mor), 3.905 (m, 4H, C*H_2_*-2 and C*H_2_*-6 Mor), 7.409 (ddd, 1H, J 8.2, 7.0, 1.1, C*H*-6), 7.716 (ddd, 1H, J 8.3, 7.0, 1.3, C*H*-7), 7.847 (dd, 1H, J 8.3, 0.9, C*H*-5), 7.880 (bd, 1H, J 8.4, C*H*-8); ^13^C NMR (CDCl_3_) 25.16 (*C*H_3_), 50.26 (*C*H_2_-3 and *C*H_2_-5 Mor), 66.80 (*C*H_2_-3 and *C*H_2_-5 Mor), 114.36 (*C*_q_-4a), 124.61 (*C*H-5), 124.80 (*C*H-6), 127.68 (*C*H-8), 132.68 (*C*H-7), 151.68 (*C*_q_-8a), 163.04 (*C*_q_-2), 164.63 (*C*_q_-4).

*4-(3,4-dihydroisoquinolin-2-yl)-2-methyl-quinazoline:* Conditions: ether, rt, 18 h; 93% yield (overall from two steps); R_f_ 0.34 (EtOAc:hexane 1:1); m. p. 107.1–107.3 °C; ^1^H NMR (CDCl_3_) 2.704 (s, 3H, C*H_3_*), 3.175 (t, 2H, J 5.8, C*H_2_*-7 THQ), 4.027 (t, 2H, J 5.8, C*H_2_*-8 THQ), 4.928 (s, 2H, C*H_2_*-2 THQ), 7.20–7.22 (m, 4H, C*H*-3-C*H*-6 THQ), 7.405 (ddd, 1H, J 8.2, 6.9, 1.2, C*H*-6), 7.701 (ddd, 1H, J 8.3, 6.9, 1.4, C*H*-7), 7.842 (bd, 1H, J 8.3, C*H*-8), 7.935 (dd, 1H, J 8.3, 1.1, C*H*-5); ^13^C NMR (CDCl_3_) 26.36 (*C*H_3_), 29.09 (*C*H_2_-7 THQ), 48.34 (*C*H_2_-8 THQ), 51.16 (*C*H_2_-2 THQ), 114.55 (*C*_q_-4a), 124.41 (*C*H-6), 124.80 (*C*H-5), 126.36, 126.60, 126.72, 128.81 (*C*H-3-*C*H-6 THQ), 127.65 (*C*H-8), 132.41 (*C*H-7), 133.92 (*C*_q_-2a THQ), 134.68 (*C*_q_-6a THQ), 151.97 (*C*_q_-8a), 163.03 (*C*_q_-2), 164.12 (*C*_q_-4); HR-MS(ESI^+^)*m*/*z* calcd. for C_18_H_18_N_3_^+^ [M + H]^+^ 276.1495, found 276.1488, ∆ = −0.7 mDa.

*2-methyl-N-(pyridin-2-yl)quinazolin-4-amine:* Conditions: DCE, 100 °C in a closed vessel, 6 h; 43% yield (overall from two steps); R_f_ 0.18 (5% MeOH in DCM); m. p. 111.7–111.9 °C; the compound NMR spectra show a slow exchange between different sites at room temperature, and so the chemical shifts for CH carbon signals are extracted from the HSQC experiment. ^1^H NMR (CDCl_3_) 7.046 (bs, 1H), 7.518 (bs, 1H), 7.790 (bs, 2H), 7.861 (bs, 1H), 7.961 (bs, 1H), 8.345 (bs, 1H), 8.827 (bs, 1H), 15.191 (bs, 1H, N*H*); ^13^C NMR (CDCl_3_) 114.45 (*C*H), 118.96 (*C*H), 120.45 (*C*H), 126.14 (*C*H), 128.23 (*C*H), 133.09 (*C*H), 138.26 (*C*H), 145.50 (*C*H), 147.76; HR-MS(ESI^+^)*m*/*z* calcd. for C_14_H_13_N_3_^+^ [M + H]^+^ 237.1135, found 237.1130, ∆ = −0.5 mDa.

*2-methyl-N-(quinolin-8-yl)quinazolin-4-amine:* Conditions: DCE, 100 °C in a closed vessel, 5 h; 71% yield (overall from two steps); R_f_ 0.35 (5% MeOH in DCM); m. p. 228.2–228.6 °C; ^1^H NMR (CDCl_3_) 3.009 (s, 3H, C*H_3_*), 7.584 (dd, 1H, J 8.2, 4.2, C*H*-3 AQ), 7.65–7.69 (m, 2H, C*H*-5 and C*H*-6 AQ), 7.745 (bt, 1H, J 7.6, C*H*-6), 7.930 (bt, 1H, J 7.6, C*H*-7), 8.256 (d, 1H, J 8.2, C*H*-5), 8.276 (dd, 1H, J 8.2, 1.4, C*H*-4 AQ), 8.404 (bd, 1H, J 7.4, C*H*-8), 8.955 (dd, 1H, J 4.2,1.5, C*H*-2 AQ), 9.270 (dd, 1H, J 6.9, 1.9, C*H*-7 8-AQ), 11.382 (bs, 1H, N*H*); ^13^C NMR (CDCl_3_) 24.15 (*C*H_3_), 113.23 (*C*_q_-4a), 118.27 (*C*H-7 AQ), 121.30 (*C*H-5), 122.23 (*C*H-3 AQ), 123.02 (*C*H-6 AQ), 123.80 (*C*H-8), 127.38 (*C*H-5 AQ), 127.96 (*C*_q_-4a AQ), 128.05 (*C*H-6), 133.20 (*C*_q_-8 AQ), 134.95 (*C*H-7), 136.84 (*C*H-4 AQ), 139.06 (*C*_q_-8a AQ), 148.78 (*C*H-2 AQ), 157.18 (*C*_q_-4), 163.02 (*C*_q_-2); HR-MS(ESI^+^)*m*/*z* calcd. for C_18_H_15_N_4_^+^ [M + H]^+^ 287.1291, found 287.1285, ∆ = −0.6 mDa.

*4-(piperidin-1-yl)-2-(quinolin-2-yl)quinazoline:* Conditions: benzene, rt, 16 h; 62% yield (overall from two steps); R_f_ 0.28 (DCM:MeOH:NH_4_OH 100:3:1); m. p. 139.6–139.9 °C; ^1^H NMR (CDCl_3_) 1.800 (m, 2H, C*H_2_*-4 Pip), 1.858 (m, 4H, C*H_2_*-3 and C*H_2_*-5 Pip), 3.868 (m, 4H, C*H_2_*-2 and C*H_2_*-6 Pip), 7.448 (ddd, 1H, J 8.3, 6.9, 1.1, C*H*-6), 7.556 (ddd, 1H, J 8.0, 6.9, 1.1, C*H*-6 Q), 7.72–7.76 (m, 2H, C*H*-7 and C*H*-7 Q), 7.845 (dd, 1H, J 8.1, 1.0, C*H*-5 Q), 7.909 (dd, 1H, J 8.3, 0.8, C*H*-5), 8.227 (dd, 1H, J 8.5, 0.5, C*H*-8), 8.292 (dd, 1H, J 8.5, 0.5, C*H*-4 Q), 8.435 (d, 1H, J 8.5, C*H*-8 Q), 8.699 (d, 1H, J 8.5, C*H*-3 Q); ^13^C NMR (CDCl_3_) 24.86 (*C*H_2_-4 Pip), 26.06 (*C*H_2_-3 and *C*H_2_-5 Pip), 51.02 (*C*H_2_-2 and *C*H_2_-6 Pip), 115.77 (*C*_q_-4a), 121.29 (*C*H-3 Q), 124.95 (*C*H-5), 125.46 (*C*H-6), 127.08 (*C*H-6 Q), 127.32 (*C*H-5 Q), 128.43 (*C*_q_-4a Q), 129.32 (*C*H-7 Q), 129.93 (*C*H-8), 131.01 (*C*H-8 Q), 132.31 (*C*H-7), 136.65 (*C*H-4 Q), 148.24 (*C*_q_-8a Q), 152.80 (*C*_q_-8a), 156.02 (*C*_q_-2 Q), 158.45 (*C*_q_-2), 165.22 (*C*_q_-4); HR-MS(ESI^+^)*m*/*z* calcd. for C_22_H_21_N_4_^+^ [M + H]^+^ 341.1761, found 341.1754, ∆ = −0.7 mDa.

*4-(morpholin-4-yl)-2-(quinolin-2-yl)quinazoline:* Conditions: benzene, rt, 17 h; 52% yield (overall from two steps); R_f_ 0.17 (EtOAc); m. p. 266.7–266.9 °C as HCl salt; ^1^H NMR (CDCl_3_) 3.904 (m, 4H, C*H_2_*-3 and C*H_2_*-5 Mor), 3.950 (m, 4H, C*H_2_*-2 and C*H_2_*-6 Mor), 7.470 (ddd, 1H, J 8.2, 6.9, 1.2, C*H*-6), 7.555 (ddd, 1H, J 8.0, 6.8, 1.4, C*H*-6 Q), 7.741 (ddd, 1H, J 8.4, 6.8, 1.4, C*H*-7 Q), 7.774 (ddd, 1H, J 8.3, 6.9, 1.4, C*H*-7), 7.838 (bd, 1H, J 8.1, C*H*-5 Q), 7.903 (dd, 1H, J 8.4, 0.9, C*H*-5), 8.253 (dd, 1H, J 8.4, 0.8, C*H*-8), 8.283 (bd, 1H, J 8.5, C*H*-4 Q), 8.430 (dd, 1H, J 8.4, 0.5, C*H*-8 Q), 8.658 (d, 1H, J 8.6, C*H*-3 Q); ^13^C NMR (CDCl_3_) 50.32 (*C*H_2_-3 and *C*H_2_-5 Mor), 66.79 (*C*H_2_-2 and *C*H_2_-6 Mor), 115.60 (*C*_q_-4a), 121.24 (*C*H-3 Q), 124.51 (*C*H-5), 125.98 (*C*H-6), 127.20 (*C*H-6 Q), 127.36 (*C*H-5 Q), 128.32 (*C*_q_-4a Q), 129.45 (*C*H-7 Q), 130.25 (*C*H-8), 130.89 (*C*H-8 Q), 132.64 (*C*H-7), 136.73 (*C*H-4 Q), 148.22 (*C*_q_-8a Q), 152.91 (*C*_q_-8a), 155.74 (*C*_q_-2 Q), 158.45 (*C*_q_-2), 165.07 (*C*_q_-4); HR-MS(ESI^+^)*m*/*z* calcd. for C_21_H_19_N_4_O^+^ [M + H]^+^ 343.1553, found 343.1547, ∆ = −0.6 mDa.

*4-(3,4-dihydroisoquinolin-2-yl)-2-(quinolin-2-yl)quinazoline:* Conditions: benzene, rt, 16 h; 71% yield (overall from two steps); R_f_ 0.48 (EtOAc); m. p. 144.4–144.6 °C; ^1^H NMR (CDCl_3_) 3.251 (t, 2H, J 5.8, C*H_2_*-7 THQ), 4.201 (t, 2H, J 5.8, C*H_2_*-8 THQ), 5.141 (s, 2H, C*H_2_*-2 THQ), 7.25–7.26 (m, 4H, C*H*-3–C*H*-6 THQ), 7.517 (ddd, 1H, J 8.0, 6.9, 1.1, C*H*-6), 7.858 (ddd, 1H, J 8.0, 6.9, 1.1, C*H*-6 Q), 7.757 (ddd, 1H, J 8.5, 6.9, 1.3, C*H*-7 Q), 7.798 (ddd, 1H, J 8.3, 7.0, 1.2, C*H*-7), 7.880 (dd, 1H, J 8.1, 0.8, C*H*-5 Q), 8.047 (dd, 1H, J 8.3, 0.7, C*H*-5), 8.305 (bd, 1H, J 8.4, C*H*-8), 8.343 (d, 1H, J 8.5, C*H*-4 Q), 8.457 (d, 1H, J 8.6, C*H*-8 Q), 8.738 (d, 1H, J 8.5, C*H*-3 Q); ^13^C NMR (CDCl_3_) 27.18 (*C*H_2_-7 THQ), 46.44 (*C*H_2_-8 THQ), 49.33 (*C*H_2_-2 THQ), 113.62 (*C*_q_-4a), 119.30 (*C*H-3 Q), 122.83 (*C*H-5), 123.77 (*C*H-6), 124.53, 124.69, 124.91, 126.85 (*C*H-3–*C*H-6 THQ), 125.30 (*C*H-6 Q), 125.40 (*C*H-5 Q), 126.56 (*C*_q_-4a Q), 127.51 (*C*H-7 Q), 127.90 (*C*H-8), 129.11 (*C*H-8 Q), 130.66 (*C*H-7), 131.84 (*C*_q_-2a THQ), 132.77 (*C*_q_-6a THQ), 134.84 (*C*H-4 Q), 146.30 (*C*_q_-8a Q), 150.54 (*C*_q_-8a), 153.65 (*C*_q_-2 Q), 156.30 (*C*_q_-2), 162.48 (*C*_q_-4); HR-MS(ESI^+^)*m*/*z* calcd. for C_26_H_21_N_4_^+^ [M + H]^+^ 389.1761, found 389.1756, ∆ = −0.5 mDa.

#### 4.1.3. Synthesis of Perimidines

*General procedure:* A solution of 1,8-diaminonaphthalene (2 mmol) and aldehyde (2 mmol) in EtOH (20 mL) was stirred at room temperature for 6 h. Sodium pyrosulfite (Na_2_S_2_O_5_,2 mmol) was then added, and the mixture was stirred at room temperature for 24 h. Finally, the solvent was removed in vacuo, and the residue was purified by column chromatography on silica gel by using a mobile phase with a gradient of polarity from 2% MeOH in DCM to 5% MeOH in DCM.

*2-(pyridin-2-yl)-1H-perimidine:* 18% yield; R_f_ 0.43 (1% acetone in DCM); m. p. 171.3–171.6 °C (lit. [28] 175–176 °C); ^1^H NMR (DMSO-d_6_) 6.711 (dd, 1H, J 7.3, 0.8, C*H*-2), 6.776 (dd, 1H, J 7.4, 0.7, C*H*-7), 7.013 (dd, 1H, J 8.4, 0.6, C*H*-5), 7.078 (dd, 1H, J 8.4, 0.6, C*H*-4), 7.110 (dd, 1H, J 8.2, 7.6, C*H*-6), 7.186 (dd, 1H, J 8.2, 7.4, C*H*-3), 7.633 (ddd, 1H, J 7.5, 4.8, 1.1, C*H*-5 Py), 8.017 (td, 1H, J 7.8, 1.7, C*H*-4 Py), 8.304 (dt, 1H, J 7.9, 0.9, C*H*-3 Py), 8.738 (ddd, 1H, J 4.8, 1.7, 0.9, C*H*-6 Py), 10.991 (N*H*); ^13^C NMR (DMSO-d_6_) 104.00 (*C*H-7), 114.59 (*C*H-2), 118.20 (*C*H-5), 120.26 (*C*H-4), 122.11 (*C*H-3 Py), 122.80 (*C*_q_-8a), 126.59 (*C*H-5 Py), 128.60 (*C*H-6), 129.31 (*C*H-3), 135.61 (*C*_q_-4a), 138.04 (*C*H-4 Py), 138.49 (*C*_q_-8), 145.31 (*C*_q_-1), 148.89 (*C*H-6 Py), 149.84 (*C*_q_-2 Py), 151.30 (N = *C*_q_-NH).

*2-(quinolin-2-yl)-1H-perimidine:* 42% yield; R_f_ 0.52 (DCM); m. p. 264.7–265.1 °C (lit. [29] m. p. not given); ^1^H NMR (DMSO-d_6_) 6.792 (dd, 1H, J 7.3, 0.9, C*H*-2), 6.847 (dd, 1H, J 7.4, 0.8, C*H*-7), 7.052 (bd, 1H, J 8.2, C*H*-5), 7.136 (dd, 1H, J 8.1, 0.7, C*H*-4), 7.159 (dd, 1H, J 7.9, 7.7, C*H*-6), 7.223 (dd, 1H, J 8.0, 7.4, C*H*-3), 7.737 (ddd, 1H, J 8.0, 6.9, 1.1, C*H*-6 Q), 7.904 (ddd, 1H, J 8.4, 6.9, 1.4, C*H*-7 Q), 8.110 (dd, 1H, J 8.1, 1.1, C*H*-5 Q), 8.254 (dd, 1H, J 8.5, 0.7, C*H*-8 Q), 8.410 (d, 1H, J 8.6, C*H*-3 Q), 8.560 (d, 1H, J 8.6, C*H*-4 Q), 10.748 (N*H*); ^13^C NMR (DMSO-d_6_) 104.05 (*C*H-7), 115.16 (*C*H-2), 118.31 (*C*H-5), 119.02 (*C*H-3 Q), 120.75 (*C*H-4), 122.90 (*C*_q_-8a), 128.37 (*C*H-6 Q), 128.52 (*C*H-5 Q), 128.60 (*C*H-6), 129.16 (*C*_q_-4a Q), 129.26 (*C*H-3), 129.52 (*C*H-8 Q), 130.81 (*C*H-7 Q), 135.72 (*C*_q_-4a), 137.73 (*C*H-4 Q), 138.44 (*C*_q_-8), 145.25 (*C*_q_-1), 146.75 (*C*_q_-8a Q), 150.05 (*C*_q_-2 Q), 151.40 (N = *C*_q_-NH).

*2-(imidazol-2-yl)-1H-perimidine:* 92% yield; R_f_ 0.34 (5% MeOH in DCM); m. p. 241.1–241.4 °C; ^1^H NMR (DMSO-d_6_; 353K) 6.685 (dd, 2H, J 7.4, 0.9, C*H*-2 and C*H*-7), 7.029 (bd, 2H, 7.8, C*H*-4 and C*H*-5), 7.129 (bd, 2H, 7.8, C*H*-3 and C*H*-6), 7.213 (bs, 2H, 2C*H*Im); ^13^C NMR (DMSO-d_6_; 353K) 113.42 (*C*H-2 and *C*H-7), 119.07 (*C*H-4 and (*C*H-5), 128.76 (*C*H-4 and *C*H-5 Im), 122.48 (*C*_q_-8a), 128.76 (*C*H-3 and *C*H-6), 137.83 (*C*_q_-4a), 135.71 (*C*_q_-1 and *C*_q_-8), 140.77 (*C*_q_-2 Im), 144.82 (N=*C*_q_-NH); HR-MS(ESI^+^)*m*/*z* calcd. for C_14_H_11_N_4_^+^ [M + H]^+^ 235.0978, found 235.0975, ∆ = −0.3 mDa.

### 4.2. Biological Experiments

#### 4.2.1. Cells and Cell Culture

A375, a human melanoma cell line (ATCC catalog no. CRL-1619™), has been used in all cellular experiments. The cells have been maintained in culture flasks with complete Dulbecco’s modified Eagle’s medium (DMEM) at 37 °C and 5% CO_2_ in a humidified atmosphere incubator. When cells reached 80–90% confluence, they were harvested using Trypsin/EDTA and were prepared for the following experiments.

#### 4.2.2. WST-1 Cell Proliferation Assay

WST-1 assay (Sigma-Aldrich Co., Darmstadt, Germany) was performed to assess the cytotoxicity of the heterocyclic ligands as previously described [30,31]. Briefly, the cells were seeded into 96-well plates at a density of 2 × 10^4^ cells per well and incubated for 24 h at 37 °C and 5% CO_2_. On the following day, the culture medium was replaced with fresh medium, and the cells were exposed to increasing concentrations of the tested heterocyclic ligands for another 24 h. At the end of incubation, the cell medium was aspirated, and a new medium was provided. After that, the WST-1 reagent was added directly to the cells in a ratio of 1:10 according to the manufacturer’s instructions. After 2 h incubation at 37 °C in the dark, the amount of the produced formazan by the cells was measured by absorbance at 450 nm using a standard microplate reader (Thermo Scientific Multiskan Spectrum, Waltham, MA, USA). The cell proliferation data were normalized to the percentage of the untreated control. The corresponding half-maximal inhibitory concentration (IC_50_) values were calculated using GraphPad Prism 7 (GraphPad Software, San Diego, CA, USA).

### 4.3. Computational Details

To obtain the necessary DFT [19,20] affinities of heterocyclic ligands to a model G-quadruplex, we use the hybrid long range and dispersion corrected wB97XD functional [32,33] at the 6-31G(d,p) basis set level, as implemented in the Gaussian 16 program system [34]. Default optimization criteria have been applied in Gaussian. Explicit electron correlated energies are calculated at the RI-MP2/6-31G(d,p) level using GAMESS-US [35], with an SVP auxiliary basis set [36] as single-point calculations at the optimized DFT geometries.

## 5. Conclusions

We have developed a model of the ligand with G-quadruplex interactions whereby the approximately planar heterocyclic ligand system is stacked to a plane of the quadruplex [16]. Quantum chemical DFT calculations indicate that computed ligand affinities to the G-quadruplex should correlate with ligand activities as anticancer agents. To verify this hypothesis, we have synthesized some 4-aminoquinazolines and 2-hetarylperimidines and have determined their anticancer activity quantitatively in the form of IC_50_. We have found a good linear relationship of theoretically computed DFT ligand affinities, A_L_, and log (IC_50_). This quantitative structure-activity relationship, QSAR, provides a means for the design of novel small heterocyclic G4-ligands to be tested as anticancer agents under the described putative stacking mechanism of novel drug-like heterocycles [37,38] to G-quadruplexes.

## Figures and Tables

**Table 1:** Structure of the investigated compounds, experimentally determined IC_50_ values, mol, and computed DFT wB97XD/6-31G(d,p)) and RI-MP2/6-31G(d,p)//wB97XD/6-31G(d,p) ligand affinities, kcal·mol^−1^. Full energy data are given in the Supplementary Materials.

No	Formula	IC_50_	Affinity (DFT)	Affinity (RI-MP2)
**1**	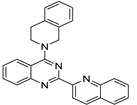	1.43 × 10^−5^	−39.04	−28.76
**2**	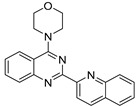	2.90 × 10^−5^	−38.08	−41.79
**3**	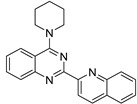	3.19 × 10^−5^	−37.63	−44.42
**4**	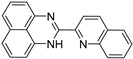	4.47 × 10^−5^	−35.02	−41.04
**5**	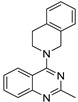	5.77 × 10^−5^	−33.03	−33.44
**6**	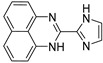	6.05 × 10^−5^	−31.51	−35.71
**7**	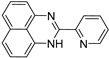	9.37 × 10^−5^	−32.08	−38.74
**8**	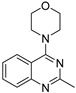	1.28 × 10^−4^	−26.64	−26.76
**9**	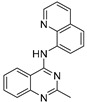	9.78 × 10^−5^	−35.23	−45.02
**10**	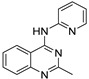	1.61 × 10^−4^	−31.29	−37.96

## Data Availability

Data are freely available in the Supplementary Material. More atomic coordinates available upon request.

## Supplementary Materials

The following supporting information can be downloaded at: https://www.mdpi.com/article/10.3390/molecules27217577/s1; Figure S1: The optimized Q2 complex of **1**; Figure S2: The optimized three-G4-layered Q3 complex of **1**; Table S1: Computed electronic energies with the 6-31G(d,p) basis set, in a.u., and ligand affinities, in kcal·mol^−1^, against experimentally determined IC_50_ values, mol; Table S2: Ligand **1**—Atomic coordinates of the optimized two-layered G4-quadruplex Q2.

## Sample Availability

Samples of the compounds are not available from the authors.
