# Supplementary material for: Small Heterocyclic Ligands as Anticancer Agents: QSAR with a Model G-Quadruplex
Source: Molecules. 2022 Nov 4;27(21):7577. doi: 10.3390/molecules27217577 (PMC9655707; doi:10.3390/molecules27217577)
Supplement: Supplementary file 1 [file molecules-27-07577-s001.zip › molecules-1945904-supplementary.pdf]

Supporting material to

# Small Heterocyclic G-quadruplex Ligands as Anticancer Agents: a QSAR

**Jose Kaneti [1], Vanya Kurteva [1], Milena Georgieva [2], Natalia Krasteva [3], George Miloshev [2], Nadezhda Tabakova [1], Zhanina Petkova [1] and Snezhana M. Bakalova [1,*]**

[1] Institute of Organic Chemistry with Centre of Phytochemistry, Bulgarian Academy of Sciences, “Acad. Georgi Bonchev” Str., Bl. 9, 1113 Sofia, Bulgaria

[2] Institute of Molecular Biology “R. Tsanev”, Bulgarian Academy of Sciences, “Acad. Georgi Bonchev” Str., Bl. 21, 1113 Sofia, Bulgaria

[3] Institute of Biophysics and Biomedical Engineering, Bulgarian Academy of Sciences, “Acad. Georgi Bonchev” Str., Bl. 21, 1113 Sofia, Bulgaria

* Correspondence: snezhana.bakalova@orgchm.bas.bg

**Table S1.** Computed electronic energies with the 6-31G(d,p) basis set, in *a.u.*, and ligand affinities, in *kcal.mol*$^{-1}$, against experimentally determined **IC$_{50}$** values, *mol*. The model quadruplex energy at wB97XD is $E_{Q2}$ = -4939.424743 *a.u.*; $E_{Q3}$ = -7709.073670 *a.u.*; $E_L$ – ligand energy; $Q_L$ – the complex ligand.quadruplex energy; $A_{QL}$: ligand affinity. Top row: wB97XD/6-31G(d,p); bottom row: RI-MP2/6-31G(d,p)//wB97XD; $E_{Q2}$ = -4927.803098 *a.u.* For some of the ligands and the respective complexes, more than one minimum has been located due to low-energy tautomerization or rotation processes.

| | **Ligands** | **$E_L$ - wB97XD/ 6-31G(d,p)** | **$Q_{L2}$ - wB97XD/ 6-31G(d,p)** | **$A_{QL2}$** | **$IC_{50}$** |
|---|---|---|---|---|---|
| 1 | 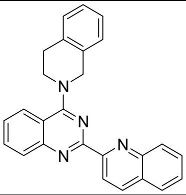 | -1221.484259<br>-1218.157251 | -6160.971211<br>-6146.025961 | **-39.04**<br>**-43.30** | **1.43 x 10$^{-5}$** |
| 2 | 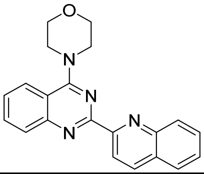 | -1104.982776<br>-1104.983076<br>-1102.014583 | -6044.467535<br>-6044.468157<br>-6029.884273 | **-37.66**<br>**-41.79** | **2.9 x 10$^{-5}$** |
| 3 | 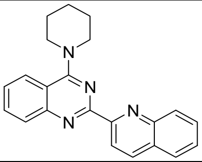 | -1069.087694<br>-1069.108354<br>-1069.113637<br>-1066.179220<br>-1066.184832 | -6008.596043 | **-37.63**<br>**-44.42** | **3.19 x 10$^{-5}$** |
| 4 | 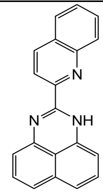 | -933.966342 | -5873.446834 | **-34.98**<br>**-41.04** | **4.47 x 10$^{-5}$** |
| **5** | 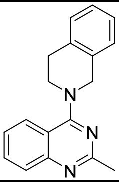 | -860.183549<br>-857.811416<br>-857.810868 | -5799.660935<br>-5785.667800 | **-33.03**<br>**-33.44** | **5.8 x 10$^{-5}$** |

| | | | | | |
|---|---|---|---|---|---|
| 6 | 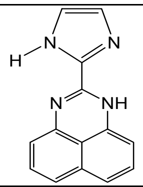 | -758.318366 | -5697.793309 | **-31.50**<br>**-35.71** | **6.05 x 10$^{-5}$** |
| 7 | 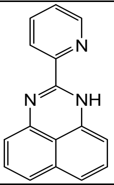 | -780.369285 | -5719.845155 | **-32.08**<br>**-38.74** | **9.37 x 10$^{-5}$** |
| 8 | 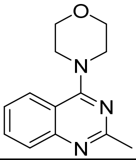 | -743.685384<br>-741.672301 | -5683.152521<br>-5683.151177<br>-5669.521223 | **-26.60**<br>**-28.76** | **1.28 x 10$^{-4}$** |
| 9 | 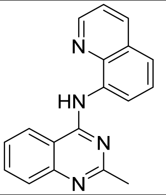 | -913.106098<br>-913.293027<br>-913.115572<br>-910.653425 | -5852.581625<br>-5852.568090<br>-5838.500144<br>-5838.493578 | **-35.20**<br>**-45.02** | **9.8 x 10$^{-5}$** |
| 10 | 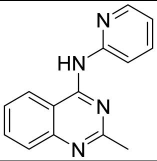 | -759.521386<br>√<br>-757.479491 | -5698.995996<br>-5698.995265 | **-31.29**<br>**-37.96** | **1.61 x 10$^{-4}$** |
| 11 | 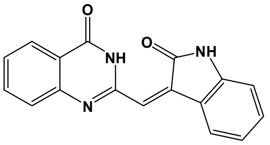<br>Schizocommunin [29] | -968.888943 | -5908.369172 | **-34.82**<br>**-40.74** | **> 5 x 10$^{-4}$** |
| 12 | 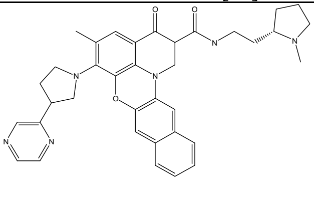<br>Quarfloxin CX3543 [42] | -2007.047343 | -6946.563341 | **-57.30**<br>**-70.67** | |

**Table S2.** Q2.Ligand 1—atomic coordinates of the two-layered G4-quadruplex complex, Gaussian format, wB97XD/6-31G(d,p).

| | | | | | |
|---|---|---|---|---|---|
| 1 | 7 | 0 | -6.998881 | -0.528519 | -1.146479 |
| 2 | 6 | 0 | -6.706627 | 0.764359 | -1.343028 |
| 3 | 6 | 0 | -5.487695 | 1.334703 | -1.673569 |
| 4 | 6 | 0 | -4.353361 | 0.487503 | -1.800931 |
| 5 | 7 | 0 | -4.681373 | -0.850984 | -1.592501 |
| 6 | 6 | 0 | -5.941179 | -1.313536 | -1.293625 |
| 7 | 7 | 0 | -7.571843 | 1.822026 | -1.254280 |
| 8 | 6 | 0 | -6.851961 | 2.958665 | -1.536506 |
| 9 | 7 | 0 | -5.599285 | 2.705124 | -1.792795 |
| 10 | 8 | 0 | -3.189199 | 0.818799 | -2.054698 |
| 11 | 7 | 0 | -6.087061 | -2.635588 | -1.136603 |
| 12 | 7 | 0 | -1.748103 | 3.137772 | -2.781453 |
| 13 | 6 | 0 | -0.407820 | 2.790311 | -2.940251 |
| 14 | 6 | 0 | 0.393494 | 3.917630 | -3.270127 |
| 15 | 6 | 0 | -0.219173 | 5.155744 | -3.381606 |
| 16 | 7 | 0 | -1.516130 | 5.462880 | -3.241113 |
| 17 | 6 | 0 | -2.254575 | 4.406420 | -2.934199 |
| 18 | 7 | 0 | 1.753380 | 4.017562 | -3.481510 |
| 19 | 6 | 0 | 1.960604 | 5.284023 | -3.710145 |
| 20 | 7 | 0 | 0.802437 | 6.024172 | -3.660679 |
| 21 | 8 | 0 | -0.037428 | 1.620808 | -2.789200 |
| 22 | 7 | 0 | -3.574374 | 4.562646 | -2.764778 |
| 23 | 7 | 0 | 2.296180 | 0.078649 | -3.243245 |
| 24 | 6 | 0 | 3.556364 | 0.531689 | -3.554208 |
| 25 | 7 | 0 | 4.620670 | -0.250052 | -3.633497 |
| 26 | 6 | 0 | 4.337106 | -1.530000 | -3.353540 |
| 27 | 6 | 0 | 3.107304 | -2.097658 | -3.059707 |
| 28 | 6 | 0 | 1.968808 | -1.251415 | -2.987231 |
| 29 | 7 | 0 | 3.232826 | -3.451542 | -2.819874 |
| 30 | 6 | 0 | 4.507051 | -3.693071 | -2.959355 |
| 31 | 7 | 0 | 5.222471 | -2.571061 | -3.294684 |
| 32 | 7 | 0 | 3.690382 | 1.844528 | -3.795145 |
| 33 | 8 | 0 | 0.802714 | -1.569549 | -2.721964 |
| 34 | 7 | 0 | -0.719341 | -3.914108 | -2.361464 |
| 35 | 6 | 0 | -2.023652 | -3.558955 | -2.033871 |
| 36 | 6 | 0 | -2.843963 | -4.700142 | -1.820768 |
| 37 | 6 | 0 | -2.269024 | -5.956884 | -1.928592 |
| 38 | 7 | 0 | -1.005035 | -6.274428 | -2.233582 |
| 39 | 6 | 0 | -0.250752 | -5.203609 | -2.439782 |
| 40 | 7 | 0 | -4.173540 | -4.794464 | -1.462391 |
| 41 | 6 | 0 | -4.398561 | -6.074450 | -1.355816 |
| 42 | 7 | 0 | -3.281675 | -6.828708 | -1.625798 |
| 43 | 8 | 0 | -2.353899 | -2.368814 | -1.949916 |
| 44 | 7 | 0 | 1.039436 | -5.368259 | -2.761578 |
| 45 | 1 | 0 | -0.070565 | -3.125170 | -2.516335 |
| 46 | 1 | 0 | 1.738067 | -4.616115 | -2.748048 |

| | | | | | |
|---|---|---|---|---|---|
| 47 | 1 | 0 | 1.363587 | -6.320156 | -2.742943 |
| 48 | 1 | 0 | 1.514415 | 0.745888 | -3.163287 |
| 49 | 1 | 0 | 4.630826 | 2.159196 | -3.958641 |
| 50 | 1 | 0 | 2.951964 | 2.538597 | -3.644662 |
| 51 | 1 | 0 | 0.701798 | 7.009700 | -3.841709 |
| 52 | 1 | 0 | -2.381166 | 2.360334 | -2.536451 |
| 53 | 1 | 0 | -3.915401 | 5.502620 | -2.866468 |
| 54 | 1 | 0 | -4.210091 | 3.835548 | -2.419122 |
| 55 | 1 | 0 | -3.901561 | -1.520793 | -1.705539 |
| 56 | 1 | 0 | -7.026326 | -2.950500 | -0.966782 |
| 57 | 1 | 0 | -5.341260 | -3.329819 | -1.265824 |
| 58 | 1 | 0 | -7.306822 | 3.937801 | -1.530852 |
| 59 | 1 | 0 | -5.341292 | -6.526524 | -1.085811 |
| 60 | 1 | 0 | 4.981073 | -4.653928 | -2.827036 |
| 61 | 1 | 0 | 2.919107 | 5.736971 | -3.915245 |
| 62 | 1 | 0 | -8.558281 | 1.758595 | -1.062052 |
| 63 | 1 | 0 | -3.214533 | -7.833390 | -1.643641 |
| 64 | 1 | 0 | 6.218777 | -2.500941 | -3.420117 |
| 65 | 19 | 0 | -0.887573 | -0.250586 | -0.980137 |
| 66 | 7 | 0 | -4.336242 | -4.434176 | 1.879209 |
| 67 | 6 | 0 | -5.033892 | -3.294051 | 1.960876 |
| 68 | 6 | 0 | -4.598727 | -1.995335 | 1.745851 |
| 69 | 6 | 0 | -3.251477 | -1.789448 | 1.342486 |
| 70 | 7 | 0 | -2.553580 | -2.990909 | 1.238167 |
| 71 | 6 | 0 | -3.079950 | -4.232025 | 1.510226 |
| 72 | 7 | 0 | -6.359873 | -3.169736 | 2.276647 |
| 73 | 6 | 0 | -6.661523 | -1.829901 | 2.235271 |
| 74 | 7 | 0 | -5.630195 | -1.095978 | 1.923806 |
| 75 | 8 | 0 | -2.691669 | -0.713192 | 1.094045 |
| 76 | 7 | 0 | -2.255300 | -5.282393 | 1.411716 |
| 77 | 7 | 0 | -3.433244 | 2.022590 | 1.013822 |
| 78 | 6 | 0 | -2.314243 | 2.712527 | 0.562473 |
| 79 | 6 | 0 | -2.574204 | 4.100609 | 0.408690 |
| 80 | 6 | 0 | -3.839879 | 4.574702 | 0.710490 |
| 81 | 7 | 0 | -4.902068 | 3.893114 | 1.155748 |
| 82 | 6 | 0 | -4.648025 | 2.599495 | 1.296703 |
| 83 | 7 | 0 | -1.737118 | 5.128830 | 0.030285 |
| 84 | 6 | 0 | -2.480154 | 6.199349 | 0.086804 |
| 85 | 7 | 0 | -3.765355 | 5.924720 | 0.491315 |
| 86 | 8 | 0 | -1.250844 | 2.119029 | 0.344721 |
| 87 | 7 | 0 | -5.619853 | 1.791382 | 1.736340 |
| 88 | 7 | 0 | 1.451378 | 2.733118 | -0.138881 |
| 89 | 6 | 0 | 2.000350 | 3.984308 | -0.293087 |
| 90 | 7 | 0 | 3.284965 | 4.204143 | -0.517094 |
| 91 | 6 | 0 | 3.991652 | 3.065582 | -0.595378 |
| 92 | 6 | 0 | 3.545881 | 1.764282 | -0.436504 |
| 93 | 6 | 0 | 2.166321 | 1.537382 | -0.192758 |
| 94 | 7 | 0 | 4.572763 | 0.865478 | -0.613558 |
| 95 | 6 | 0 | 5.616835 | 1.598159 | -0.876600 |
| 96 | 7 | 0 | 5.324089 | 2.942561 | -0.881678 |
| 97 | 7 | 0 | 1.169653 | 5.035061 | -0.185066 |

| | | | | | |
|---|---|---|---|---|---|
| 98 | 8 | 0 | 1.590912 | 0.451572 | -0.048934 |
| 99 | 7 | 0 | 2.277641 | -2.308604 | 0.113167 |
| 100 | 6 | 0 | 1.112219 | -3.048760 | 0.253126 |
| 101 | 6 | 0 | 1.381879 | -4.425144 | 0.470594 |
| 102 | 6 | 0 | 2.702628 | -4.826932 | 0.586045 |
| 103 | 7 | 0 | 3.812608 | -4.089605 | 0.482892 |
| 104 | 6 | 0 | 3.553636 | -2.815325 | 0.224403 |
| 105 | 7 | 0 | 0.522581 | -5.482323 | 0.684128 |
| 106 | 6 | 0 | 1.302298 | -6.505222 | 0.903628 |
| 107 | 7 | 0 | 2.633641 | -6.168998 | 0.856312 |
| 108 | 8 | 0 | 0.002005 | -2.501116 | 0.204088 |
| 109 | 7 | 0 | 4.570536 | -1.963664 | 0.030849 |
| 110 | 1 | 0 | 2.148461 | -1.289753 | 0.025648 |
| 111 | 1 | 0 | 4.412770 | -0.967757 | -0.143136 |
| 112 | 1 | 0 | 5.407188 | -2.160751 | 0.561774 |
| 113 | 1 | 0 | 0.436526 | 2.632039 | 0.017446 |
| 114 | 1 | 0 | 1.585819 | 5.929448 | -0.379530 |
| 115 | 1 | 0 | 0.147346 | 4.962620 | -0.164082 |
| 116 | 1 | 0 | -4.511097 | 6.585436 | 0.637463 |
| 117 | 1 | 0 | -3.296560 | 1.008608 | 1.145348 |
| 118 | 1 | 0 | -6.507016 | 2.231564 | 1.906686 |
| 119 | 1 | 0 | -5.542699 | 0.769051 | 1.790508 |
| 120 | 1 | 0 | -1.577644 | -2.913474 | 0.901746 |
| 121 | 1 | 0 | -2.682342 | -6.180255 | 1.561525 |
| 122 | 1 | 0 | -1.287413 | -5.245964 | 1.067758 |
| 123 | 1 | 0 | -7.655334 | -1.461448 | 2.441507 |
| 124 | 1 | 0 | 0.974147 | -7.515197 | 1.100275 |
| 125 | 1 | 0 | 6.613190 | 1.227176 | -1.064985 |
| 126 | 1 | 0 | -2.159885 | 7.200131 | -0.161519 |
| 127 | 1 | 0 | -6.978863 | -3.926642 | 2.517507 |
| 128 | 1 | 0 | 3.421956 | -6.775364 | 1.015135 |
| 129 | 1 | 0 | 5.971939 | 3.702070 | -1.010645 |
| 130 | 6 | 0 | 4.028207 | 0.016177 | 2.846026 |
| 131 | 6 | 0 | 2.636396 | -1.705743 | 3.310738 |
| 132 | 6 | 0 | 1.511127 | -0.846717 | 3.256482 |
| 133 | 6 | 0 | 1.824990 | 0.561387 | 3.213366 |
| 134 | 7 | 0 | 3.910247 | -1.261807 | 3.095752 |
| 135 | 6 | 0 | 2.440183 | -3.088773 | 3.531530 |
| 136 | 6 | 0 | 1.169180 | -3.600837 | 3.611117 |
| 137 | 1 | 0 | 1.019608 | -4.665347 | 3.761806 |
| 138 | 6 | 0 | 0.215077 | -1.412705 | 3.229956 |
| 139 | 6 | 0 | 0.050041 | -2.763960 | 3.423044 |
| 140 | 1 | 0 | -0.943710 | -3.197340 | 3.397626 |
| 141 | 1 | 0 | -0.643186 | -0.790322 | 2.998232 |
| 142 | 7 | 0 | 3.057538 | 0.954043 | 2.953016 |
| 143 | 1 | 0 | 3.322029 | -3.716681 | 3.590553 |
| 144 | 6 | 0 | -0.066130 | 1.367924 | 4.553146 |
| 145 | 6 | 0 | 1.291371 | 2.931427 | 3.336043 |
| 146 | 6 | 0 | -2.302223 | 4.274560 | 3.615551 |
| 147 | 6 | 0 | -1.290683 | 2.226479 | 4.366618 |
| 148 | 6 | 0 | 0.097076 | 3.816510 | 2.985754 |

| | | | | | |
|---|---|---|---|---|---|
| 149 | 1 | 0 | 1.749565 | 3.253496 | 4.284530 |
| 150 | 6 | 0 | -3.518756 | 3.891288 | 4.166974 |
| 151 | 1 | 0 | -2.215130 | 5.228418 | 3.100731 |
| 152 | 6 | 0 | -2.514272 | 1.837129 | 4.910344 |
| 153 | 6 | 0 | -1.183119 | 3.444928 | 3.693455 |
| 154 | 1 | 0 | -0.091883 | 3.743940 | 1.911776 |
| 155 | 6 | 0 | -3.629864 | 2.659009 | 4.805665 |
| 156 | 1 | 0 | -4.382422 | 4.541858 | 4.079913 |
| 157 | 1 | 0 | -2.591534 | 0.880548 | 5.421066 |
| 158 | 1 | 0 | -4.580042 | 2.341907 | 5.222294 |
| 159 | 1 | 0 | -0.348550 | 0.326441 | 4.672640 |
| 160 | 7 | 0 | 0.879764 | 1.533035 | 3.448047 |
| 161 | 1 | 0 | 2.058909 | 3.010449 | 2.568415 |
| 162 | 1 | 0 | 0.357067 | 4.861863 | 3.182313 |
| 163 | 1 | 0 | 0.436847 | 1.663472 | 5.490248 |
| 164 | 6 | 0 | 5.372460 | 0.547848 | 2.457266 |
| 165 | 6 | 0 | 5.620978 | 1.942370 | 2.586593 |
| 166 | 6 | 0 | 9.069710 | 1.992664 | 1.188024 |
| 167 | 6 | 0 | 7.462852 | 0.184118 | 1.578932 |
| 168 | 6 | 0 | 6.832132 | 2.438509 | 2.199175 |
| 169 | 1 | 0 | 4.839496 | 2.575551 | 2.985207 |
| 170 | 6 | 0 | 9.953087 | 1.093238 | 0.648314 |
| 171 | 1 | 0 | 9.327685 | 3.045665 | 1.261724 |
| 172 | 6 | 0 | 8.401096 | -0.724724 | 1.024322 |
| 173 | 6 | 0 | 7.805624 | 1.560776 | 1.661626 |
| 174 | 1 | 0 | 7.056605 | 3.497882 | 2.290801 |
| 175 | 6 | 0 | 9.614186 | -0.278923 | 0.567095 |
| 176 | 1 | 0 | 10.919681 | 1.428551 | 0.287131 |
| 177 | 1 | 0 | 8.126405 | -1.773547 | 0.982255 |
| 178 | 1 | 0 | 10.327038 | -0.979998 | 0.145311 |
| 179 | 7 | 0 | 6.258202 | -0.300766 | 1.983539 |

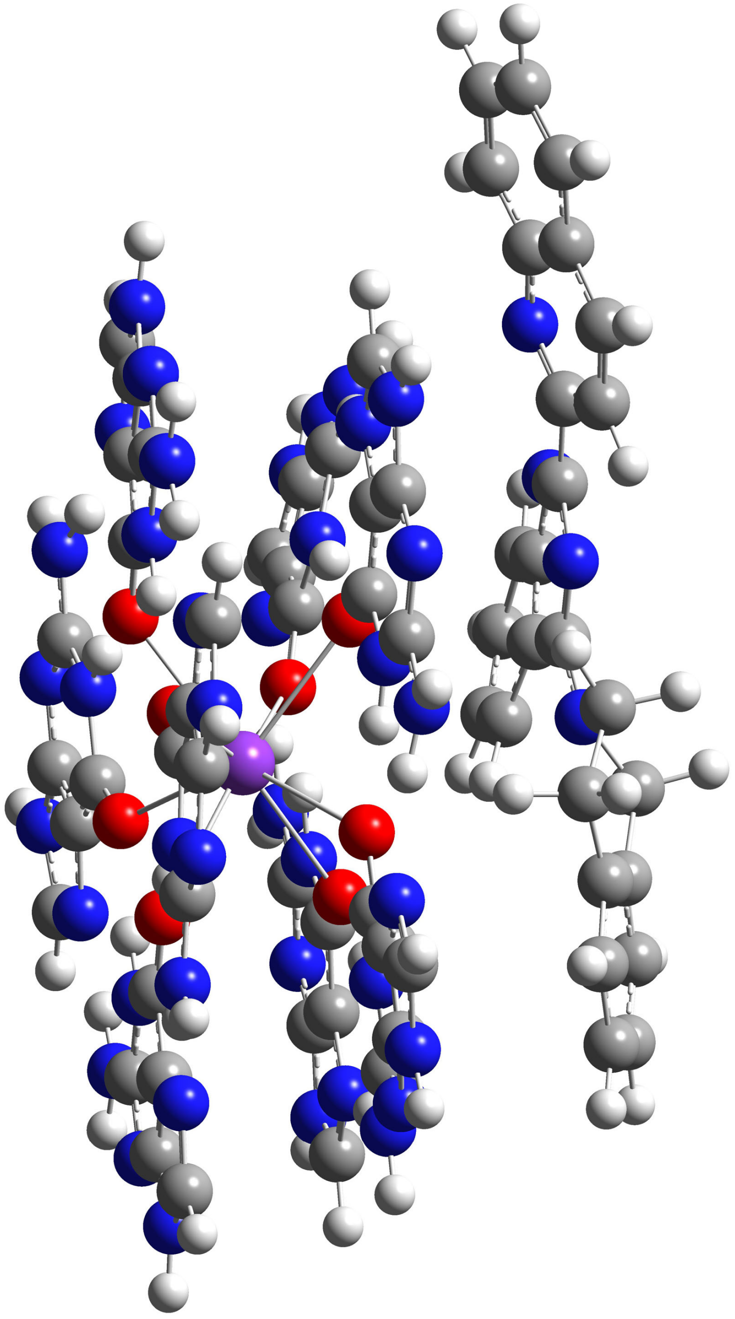

**Figure S1.** The optimized complex of the most active ligand **1**, having the lowest $IC_{50}$, "concave" form, with the model two-layered G-quadruplex. The computed stabilization energy at wB97XD/6-31G(d,p) is 39.04 kcal $mol^{-1}$.

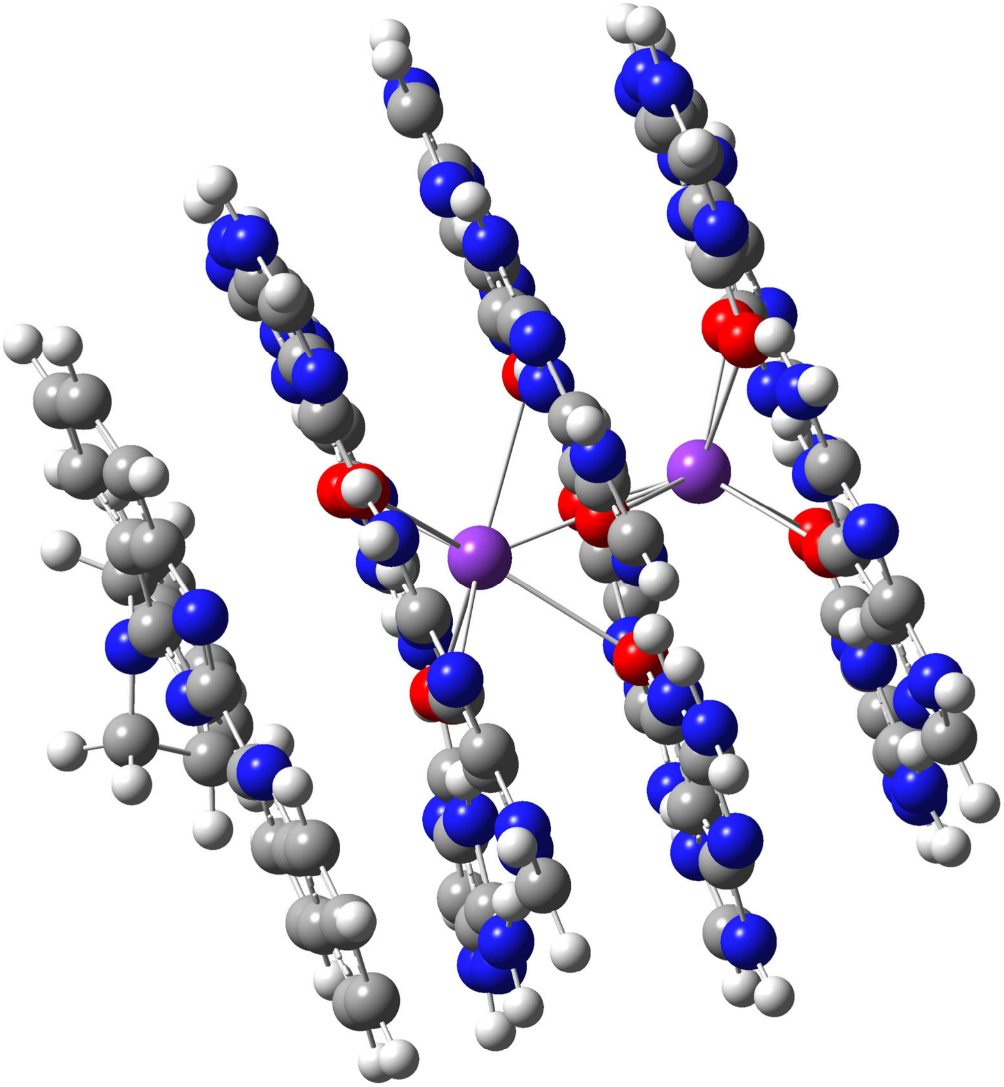

**Figure S2.** The optimized complex with the most active ligand **1**, having the lowest $IC_{50}$, "concave" form, with the model three-layered G-quadruplex. The computed stabilization energy at wB97XD/6-31G(d,p) is 41.53 kcal $mol^{-1}$.
